# Free Radicals and Neonatal Brain Injury: From Underlying Pathophysiology to Antioxidant Treatment Perspectives

**DOI:** 10.3390/antiox10122012

**Published:** 2021-12-18

**Authors:** Silvia Martini, Laura Castellini, Roberta Parladori, Vittoria Paoletti, Arianna Aceti, Luigi Corvaglia

**Affiliations:** 1 Department of Medical and Surgical Sciences, University of Bologna, 40138 Bologna, Italy; ariana.aceti2@unibo.it (A.A.); luigi.corvaglia@unibo.it (L.C.); 2 Neonatal Intensive Care Unit, IRCCS Azienda Ospedaliero-Universitaria di Bologna, 40138 Bologna, Italy; vittoria.paoletti@aosp.bo.it; 3 School of Medicine and Surgery, Alma Mater Studiorum, University of Bologna, 40126 Bologna, Italy; laura.castellini2@studio.unibo.it; 4 Specialty School of Pediatrics, Alma Mater Studiorum, University of Bologna, 40126 Bologna, Italy; roberta.parladori@gmail.com

**Keywords:** oxidative stress, free radicals, brain injury, neonate, hypoxic-ischemic encephalopathy, perinatal asphyxia, white matter injury, periventricular leukomalacia, intraventricular hemorrhage, preterm infants

## Abstract

Free radicals play a role of paramount importance in the development of neonatal brain injury. Depending on the pathophysiological mechanisms underlying free radical overproduction and upon specific neonatal characteristics, such as the GA-dependent maturation of antioxidant defenses and of cerebrovascular autoregulation, different profiles of injury have been identified. The growing evidence on the detrimental effects of free radicals on the brain tissue has led to discover not only potential biomarkers for oxidative damage, but also possible neuroprotective therapeutic approaches targeting oxidative stress. While a more extensive validation of free radical biomarkers is required before considering their use in routine neonatal practice, two important treatments endowed with antioxidant properties, such as therapeutic hypothermia and magnesium sulfate, have become part of the standard of care to reduce the risk of neonatal brain injury, and other promising therapeutic strategies are being tested in clinical trials. The implementation of currently available evidence is crucial to optimize neonatal neuroprotection and to develop individualized diagnostic and therapeutic approaches addressing oxidative brain injury, with the final aim of improving the neurological outcome of this population.

## 1. Introduction

Oxygen (O_2_) plays a key role in mitochondrial oxidative phosphorylation and in the activity of several oxidative enzymes. Under physiological conditions, more than 90% of O_2_ in the electron transport chain (ETC) is reduced to water by cytochrome oxidase, while less than 10% is reduced incompletely, leading to the formation of reactive oxygen species (ROS). ROS are characterized by an unpaired electron, which confers them oxidizing and reducing properties. Superoxide anion (O_2_^•−^) results from oxygen reduction with just one electron and it is the most common free radical in human biology [1]. Oxygen reduction with two electrons form hydrogen peroxide (H_2_O_2_), which is not a free radical but it is chemically more active than molecular oxygen and is therefore included among reactive oxygen species (ROS) [1]. Hydroxyl radical (^•^OH) is a powerful oxidant uncharged with one unpaired but extremely reactive electron; transitional metals, such as non-protein bound iron (NPBI), also contribute to ^•^OH generation through the Fenton reaction, upon H_2_O_2_ reduction. Furthermore, O_2_^•−^ interaction with nitric oxide radical (^•^NO) leads to the formation of peroxynitrite (ONOO-) and other reactive nitrogen species (RNS) [2].

ROS and RNS are intrinsically unstable molecules, able to react with membrane lipids, proteins and nucleic acids and to convert them into free radicals.

At low/moderate concentrations, free radicals are involved in several physiological processes, including mitogenic responses, modulation of the immune system, vascular regulation. For this reason, ROS and RNS levels are tightly regulated by specific antioxidant enzymes, such as catalase (CAT), superoxide dismutase (SOD) and glutathione peroxidase (GP) [1]. SOD allows the initial dismutation of O_2_^•−^ to H_2_O_2_ which, in turn, breaks down into O_2_ and water by the action of CAT and GP [3]; hence, the concerted activity of these 3 enzymes is essential for the sequential ROS catalysis. Reduced glutathione (GSH) contributes to control ROS via direct interaction or serving as a cofactor for ROS-detoxifying enzymes [4] and, as such, is considered the largest antioxidative reservoir.

The balance between ROS/RNS levels and antioxidant enzyme capacities defines the redox homeostasis and protects biological structures from oxidative damage; when this balance is shifted towards a free radical overproduction, oxidative stress develops [1]. Oxidative stress can cause harmful structural modifications (e.g., lipid peroxidation, protein carbonylation, DNA oxidation) to the cell structures, altering their function and leading to irreversible cellular damage [5].

The developing brain is particularly susceptible to oxidative stress; as such, free radicals are deeply involved in the pathophysiology of neonatal brain injury, with relevant clinical and therapeutical implications.

This narrative review aims to point out the role of free radicals in the development of neonatal brain injury in both term and preterm neonates by providing a detailed overview on the underlying pathophysiological mechanisms, currently available oxidative biomarkers and the novel antioxidant therapeutic options which have been explored in clinical settings so far.

## 2. Pathophysiological Mechanisms of Oxidative Brain Damage

The pathogenesis of oxidative brain injury relies on the interaction between the intrinsic vulnerability of the neonatal brain and the mechanisms of damage described below, which ultimately converge on free radical production. In clinical settings, these pathophysiological pathways often coexist. For instance, the sepsis-related microvascular damage can further worsen the noxious effects of infection-inflammation by triggering hypoxic-ischemic pathways, while intermittent or chronic hypoxia may be associated with a non-infectious tissue inflammation that contributes to oxidative stress. Moreover, following an intraventricular or intracranial bleeding, the oxidative damage triggered by the release of free iron can be further enhanced by the concurrent perilesional inflammation, thus contributing to the development of secondary brain injury.

Maturation-dependent neonatal characteristics, such as gestational age (GA), further modulate the impact of free radicals on the developing brain and in determining the type and the extent of oxidative brain damage. At lower GAs, the immaturity of the antioxidant enzyme systems further worsens the burden of oxidative stress [6]. Of note, data from autopsy white matter specimens of fetal brains has demonstrated a developmental mismatch in the expression of the main antioxidant enzymes, with SOD expression lagging behind that of CAT and GP up to near-term age [3]. Furthermore, during the developmental phases of central nervous system, premyelinating oligodendrocytes (pre-OL) are particularly vulnerable to the effects of free radicals, which have been shown to disrupt their maturation and differentiation into mature oligodendrocytes, thus leading to white matter injury [7].

Pre-OL death or dysmaturation is the main pathogenetic abnormality underlying the development of periventricular leukomalacia (PVL), a type of neonatal brain injury particularly frequent at lower GAs, characterized by focal necrotic and apoptotic phenomena in the periventricular white matter. In cystic PVL, the focal injury is macroscopic and tends to evolve to the formation of confluent cystic cavities, visible on cranial ultrasonography. On the other hand, in non-cystic PVL, the white matter abnormalities are microscopic and are characterized by pre-OL loss and marked astrogliosis and microgliosis, which tend to evolve in glial scars [7]. This latter form accounts for the majority of PVL cases. Cranial ultrasound may show persistent periventricular flares; however, advanced neuroimaging, such as brain magnetic resonance imaging (MRI), is required for an appropriate diagnosis [8].

**Figure 1:**
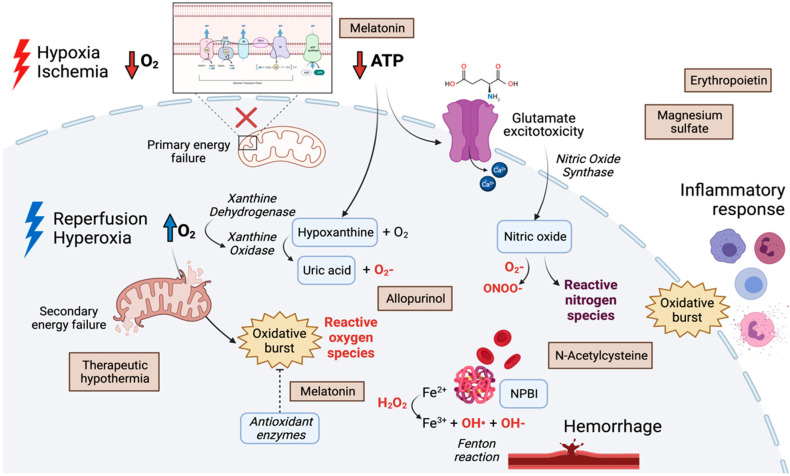
Graphical illustration of the main pathophysiological mechanisms underlying the development of oxidative brain injury in the neonatal population, namely hypoxia-ischemia-reperfusion, hypoxia-hyperoxia, inflammation and hemorrhagic insults. Pro- and antioxidant enzymes are highlighted in italics. Reactive oxygen species are marked in red. The chemical compounds in the blue-circled shapes have also been explored as oxidative biomarkers. The antioxidative treatment approaches discussed in this review (brown boxes) are also included, in correspondence of their proposed mechanisms of action. Created with BioRender.com^®^.

The downstream consequences of oxidative stress on the neonatal brain result from the complex interplay between the underlying pathophysiology, the intensity and duration of oxidative stress, the individual response to oxidative stress and the developmental stage of the central nervous system (CNS) at the time of injury. The main mechanisms leading to oxidative brain injury in the neonatal population are discussed in the following paragraphs and illustrated in Figure 1.

### 2.1. Hypoxia-Ischemia-Reperfusion

Cerebral hypoxia-ischemia is a leading pathophysiological mechanism underlying neonatal brain injury and is characterized by an acute or subacute interruption of cerebral blood flow and of the subsequent oxygen delivery. The lack of O_2_ in the mitochondrial ETC affects the oxidative phosphorylation, shifting the cell metabolism to anaerobic; the use of glucose for anaerobic glycolysis, however, is not only highly inefficient but also contributes to the depletion of cerebral glucose, which is the primary energy source for neural cells [9]. As a result of the decreased adenosine triphosphate (ATP) production, the ATP-dependent ion pumps on cell membranes become inactive, leading to intracellular accumulation of sodium and water, cell swelling and necrotic cell death. The cell membrane depolarization occurring in this phase triggers the release of glutamate, an excitatory neurotransmitter that activates apoptotic pathways via glutamate and N-methyl-D-aspartate (NMDA) receptors, and also upregulates constitutive and inducible nitric oxide synthase (NOS) to increase cerebral blood flow via NO-mediated vasodilation [10]. By enhancing free radical production, this process, defined as glutamate excitotoxicity, further contributes to the establishment of neural injury [11].

Despite the reperfusion of the ischemic area is of paramount importance, this process is key to the development of oxidative brain damage [12]. The increased O_2_ availability that follows blood flow restoration in the oxygen-depleted tissue fuels the production of free radicals by complex I and III of the mitochondrial ETC and other pro-oxidant enzymes [13]. An example is provided by the proteolytic conversion of xanthine dehydrogenase (XD) to xanthine oxidase (XO) [14], boosted by the intracellular influx of calcium in energy-depleted cells: this enzyme, using xanthine or hypoxanthine as reaction substrates and O_2_ as a cofactor, generates uric acid and O_2_^•−^, thus further enhancing intracellular ROS production [15]. The free radical burst that follows reperfusion further contributes to impair mitochondrial phosphorylation, feeding a vicious circle that finally results in secondary energy failure and programmed neuronal death [16]. This phase may last up to 72 h following the ischemic insult and represents an important therapeutic window for relevant neuroprotective strategies (e.g., therapeutic hypothermia (TH)) [17].

The pathognomonic example of hypoxia-ischemia-reperfusion brain injury in the neonatal population is the development of hypoxic-ischemic encephalopathy (HIE) following perinatal asphyxia, which still represents a leading cause of mortality and long-term neurological disability among term and near-term neonates. Following an acute ischemic insult in term infants, the deep grey nuclei are commonly involved [11]. This is ascribable to the presence of NOS-expressing striatal neurons, that are relatively resistant to hypoxia-ischemia injury and glutamate excitotoxicity [18] and, in response to the ischemic event, produce excessive amounts of RNS [19], exerting extensive nitrosative effects on the adjacent neural structures and thus further contributing to their damage.

When occurring in preterm or late-preterm neonates, the devastating effects of hypoxia-ischemia are even more enhanced, due to the inefficient antioxidant activities and to the anatomical immaturity of the CNS, which includes an increased permeability of the blood-brain-barrier (BBB) [20]. Hypoxic-ischemic injury in the preterm population more often involves white matter watershed areas. The higher susceptibility of these regions can be explained by the peculiar cerebrovascular anatomy of premature infants, characterized by the presence of arterial border- and end-zones that lay between the penetrating branches of the middle, anterior and posterior cerebral arteries [8]. As such, these areas are particularly sensible to the cerebral blood flow fluctuations that often occur in preterm neonates, as a consequence of their immature cerebrovascular autoregulation [21].

### 2.2. Intermittent Hypoxia

Due to the immaturity of the physiological mechanisms involved in respiratory control and oxygenation, along with the increased metabolic oxygen consumption, intermittent episodes of hypoxia are very frequent in premature neonates. Intermittent hypoxia is included among the pathological circumstances that can lead to oxidative stress [22].

Although the oxidative stress pathways resemble those previously described for hypoxia-ischemia reperfusion, the underlying pathophysiological mechanisms may be slightly different. First, the decreased O_2_ availability at the brain level is mainly driven by hypoxemia rather than ischemia, although a transient cerebral hypoperfusion may occur if a marked bradycardia accompanies the hypoxic spell [23]; as such, the hypoxic burden is usually less profound. Second, the restoration of an adequate O_2_ delivery to the tissue often entails an increase in inspired O_2_, leading to a transient hyperoxic phase that remarkably enhances the oxidative burst [24]. Third, the repeated alternance between hypoxia-hyperoxia resulting from tightly clustered hypoxic spells plays a key role in enhancing ROS production following these intermittent events in the preterm population. In this regard, multiple hypoxic episodes occurring 1 to 20 min apart have been associated with an increased risk of retinopathy of prematurity, whose pathophysiology includes oxidative mechanisms [25], and a duration > 1 min has been shown to significantly increase the risk of neurodevelopmental impairment [26].

The association between intermittent hypoxia and the development of brain injury, and in particular of white matter abnormalities, has been described in neonatal rat models [27,28]. Although evidence from human cohorts is not available yet, the involvement of cerebral white matter is consistent with the vulnerability of pre-OL to oxidative insults observed in preterm neonates. In particular, oxidative stress reduces the expression of differentiation-promoting genes in pre-OL and increases the expression of differentiation-inhibiting genes, finally disrupting their maturation; moreover, under conditions of oxidative stress, the global histone acetylation persists, further hindering the differentiation of pre-OL [29]. Caffeine is widely used to reduce the hypoxic burden due to the occurrence of apneic spells in the preterm population; evidence of enhanced myelination in treated rat pups [30] and of improved white matter micro-structure in preterm infants undergoing early caffeine treatment [31] further confirms the detrimental role of intermittent hypoxia on white matter development.

### 2.3. Inflammation

Inflammation-related oxidative stress plays an important role in several fetal-neonatal pathological processes, including the development of brain injury. During inflammatory responses, either triggered by underlying infections or non-infectious stimuli, the activation of immune cells such as neutrophils, macrophages and lymphocytes releases large amounts of ROS, RNS, cytokines and proteases that directly or indirectly contribute to generate oxidative stress in the involved tissues [32].

Inflammation-induced oxidative stress represents a leading mechanism underlying the development of PVL, either cystic or not. Evidence from autopsy neonatal brain samples and animal studies has shown a significant activation of white matter microglia and a remarkable increase in the concentration of such inflammatory cytokines as interferon-γ, tumor necrosis factor-α, interleukin-6 and interleukin-2 [33,34,35,36,37] in the brain tissue affected by PVL, with increased levels of protein nitration and lipid peroxidation in pre-OL [38].

The crucial role of the inflammatory microglial response and of the ensuing ROS and RNS overproduction in PVL development is further supported by the wide amount of evidence on the association between maternal chorioamnionitis, significantly higher levels of both inflammatory cytokines and oxidative biomarkers in the amniotic fluid and in cord blood [39,40,41,42,43], and subsequent PVL development [42,43,44,45,46]. On a similar note, consistently with pre-OL vulnerability to free radicals, a higher incidence of white matter abnormalities has been observed following postnatal infections [47], necrotizing enterocolitis [48] and other pro-inflammatory non-infectious conditions, such as bronchopulmonary dysplasia [49] and maternal pre-eclampsia [50], that have been largely associated with systemic inflammation and oxidative stress.

### 2.4. Hemorrhage (Hemoglobin-Induced Oxidative Damage)

Hemoglobin is a pentacoordinate hemoprotein primarily involved in O_2_ transport and delivery to biological tissues. Due to the pro-oxidant characteristics of the iron-containing heme prosthetic group, hemoglobin can also act as a powerful redox enzyme. In the presence of a superoxide anion-generating system (e.g., hypoxanthine and xanthine oxidase), the reaction between the ferrous heme iron and H_2_O_2_ promotes the formation of hydroxyl-radicals that enhance the peroxidation of membrane lipids. Hence, by acting as a Fenton reagent, free hemoglobin may catalyze hydroxyl-radical generation, subsequently contributing to generate oxidative stress [51,52]. The oxidative processes triggered by ferrous iron are also involved in ferroptosis, an additional mechanism of ROS-dependent programmed cell death, distinct from apoptosis, that has been noted after hemorrhagic events. Ferroptosis is triggered by the failure of GSH-dependent antioxidant enzymes, following which an excessive accumulation of lipid hydroperoxides occurs, leading to mitochondrial shrinkage and subsequent cell death [53,54].

A fitting example for this pathogenic mechanism in the neonatal population is provided by intraventricular hemorrhage (IVH). Due to the intrinsic fragility of the immature germinal matrix vasculature combined with the hemodynamic disturbances that characterize postnatal transition, this type of brain lesion is particularly frequent in preterm infants during the first week of life and, in severe cases, it is often associated with the development of periventricular white matter injury [55]. Following the initial bleeding and the ensuing primary brain injury, the degradation of blood components releases large amounts of hemoglobin, free iron and other neurotoxic substances, that cross the BBB and trigger perilesional inflammatory reactions and free radical production, leading to neuronal and glial apoptosis [53] and disrupting the maturation of pre-OL [56]. Furthermore, pre-OL are particularly rich in iron, which supports their differentiation [57]; as a result, these cells are particularly vulnerable to the iron-related mechanisms of oxidative damage.

This event cascade is further supported by the evidence, in neonatal rat models of IVH, of increased ROS and RNS levels in the periventricular white matter adjacent to the ventricles involved by the hemorrhage [58,59] and of impaired mitochondrial function in pre-OL exposed to hemoglobin-induced oxidative stress [60].

## 3. Free Radical Biomarkers in Neonatal Brain Injury

Free radical biomarkers are molecular metabolites of the oxidative or nitrosative reactions that can be quantitively measured in such biological fluids as plasma, urine, cerebro-spinal fluid (CSF), in order to estimate the burden of oxidative stress and the related impact on the involved tissues. Furthermore, the use of oxidative biomarkers may provide potentially useful information to monitor the efficacy of feasible treatments following hypoxic-ischemic insults, or in the context of inflammatory processes.

Gas chromatography coupled to tandem mass spectrometry is the gold standard method for the assessment of oxidant processes in biological fluids [61] and, together with other technologies such as the thiobarbituric acid method [62,63,64], has been long used to measure oxidative metabolites [65]. The recent development of high- or ultra-performance liquid chromatography has allowed to assess the concentration of ROS, RNS and of their metabolites on very small amounts of biological fluids [66], which is of paramount importance to implement the assessment of oxidative biomarkers even in neonatal settings. Due to the high costs, technical complexity and need for trained personnel associated with these techniques, however, their use is mainly limited to academic settings with research facilities [61]. Despite encouraging results, current literature on free radical biomarkers in neonates at neurological risk is often based on small sample cohorts, which represent a major limitation for the validation of these biomarkers in clinical settings and is likely due to the restricted availability of the techniques required for their assessment. Hence, further trials on bigger samples along with the development of easily and largely available analytical methods are warranted to implement the usefulness of free radical biomarkers in routine clinical practice.

The main biomarkers that have been currently proposed for the estimation of oxidative-mediated brain injury development in the neonatal population are reviewed in the following paragraphs. A brief summary of the main evidence, with particular regard to HIE and to white matter injury or IVH, is provided in Table 1 and Table 2, respectively.

### 3.1. Lipid Peroxidation Biomarkers

Due to the rich lipid composition of the brain, the assessment of lipid peroxidation products on serum, urines and, in particular, in the CSF may provide potentially useful information on the extent of cerebral oxidative injury in such at-risk groups as asphyxiated neonates or preterm infants.

Malondialdehyde (MDA) is a n-3 and n-6 fatty acid peroxidation biomarker that can be easily determined using thiobarbituric acid assays; hence, it has been long investigated in clinical settings. Increased MDA levels have been observed in the cord blood of severely asphyxiated term infants [63,67] and in the serum of newborns with HIE within the first 72 h of life [67,68,69,70,71,72] compared with healthy term neonates, and correlated with HIE severity in a linear fashion [63,68,71]. Highest MDA levels were also observed in HIE infants who died [67,69], had evidence of brain lesions at neuroimaging [73] or developed a persistent neurological impairment [69,74]. Due to its hydrophile characteristics, MDA is excreted in urine and the ratio between urinary MDA and creatinine has also been assessed. Over the first 48 h of life, this ratio was significantly increased in asphyxiated term neonates [62,63,64], especially in non-survivors [62] or in those with a higher Sarnat stage, which defines HIE severity [75].

With regard to prematurity-related brain injury, a significant increase in the concentration of plasma MDA was reported during the first 12 h of life in association with IVH development [76], whereas the evaluation of urine MDA in preterm infants developing oxygen radical diseases, including IVH/PVL, has led to controversial results [77,78]. In these latter studies, however, the specific correlation between MDA levels and prematurity-related brain lesions, however, was not investigated.

Isoprostanes are prostaglandin-like compounds derived from the free radical-catalyzed peroxidation of long-chain polyunsaturated fatty acids. Following the development of an ultra-performance liquid chromatography sensitive to very small amounts of serum [66], these compounds have been progressively investigated also in the neonatal population.

Higher levels of total isoprostanes, including 8-isoprostane, were detected in the cord blood of acidotic and depressed term infants compared to healthy neonates [79]; a positive correlation between 8-isoprostane levels and the severity of perinatal asphyxia, defined according to cord gas pH, Apgar score and neurological status at birth was also reported. However, in a recent study, serial measurements of serum isoprostanes over the first 5 days in HIE infants failed to identify neonates who developed a more severe encephalopathy and did not correlate with global brain damage severity at MRI [80].

Plasma F2-isoprostanes in extremely preterm infants aged 24–48 h were found to correlate positively with the severity of white matter injury at term MRI, assessed using validated score; this correlation was confirmed even after adjustment for GA and IVH severity [81]. Postnatal 8-isoprostane levels have also been evaluated by Ahola et al. in extremely low birth weight neonates, showing significantly higher levels on day 3 and 7 in infants with severe IVH compared to no IVH, and on day 7 in infants who developed PVL compared to those who did not [82]. In a similar fashion, increased 8-isoprostane levels were observed within the first 48 h in preterm infants with oxygen radical diseases, including IVH/PVL [78]; however, as per MDA, these conditions were not assessed separately from the other diseases.

A possible limitation of serum and urinary assessments of lipid peroxidation products is the lack of specificity for the cerebral tissue. Hence, CSF specimens may be more specific to assess the burden of cerebral oxidative stress, although their collection requires an invasive maneuver. CSF data from the neonatal population, however, are very limited. Significantly higher CSF levels of MDA within the first 72 h have been reported in asphyxiated term infants who developed HIE compared to controls [69,83], and in those who died or developed significant neurological deficits [69]. MDA levels in CSF, however, were much lower than plasma levels and showed smaller surges, resulting in a paradoxically diminished ratio between CSF and plasma MDA for increasing HIE stages [83]. As for white matter injury in the preterm population, a trend toward higher CSF levels of 8-isoprostane, but not of CSF MDA, was observed in a small series of cases compared to controls [84]; being based on a very little cohort, these data need to be evaluated in larger trials.

### 3.2. Protein Oxidation Markers

The effects of ROS and RNS on biological proteins include carbonylation, fragmentation, nitration, cross-linking and loss of thiol groups. Among the ensuing oxidation products, protein carbonyls (PC) and advanced oxidation protein products (AOPP) have been investigated as potential oxidative biomarkers in perinatal diseases [67,85].

Increased serum PC levels were seen at birth and after 48 h from the hypoxic-ischemic insult in asphyxiated term neonates, with higher levels at 48 h in those infants who developed seizures compared to those who did not [67]. On the other hand, serum concentration of AOPP in term infants aged 1 and 5 days did not differ significantly between HIE cases and healthy controls [68].

With regard to the preterm population, significantly increased blood levels in cord samples [86] and at 7 days of life [87] were observed in hypoxic compared to normoxic infants, where hypoxia was defined by an Apgar score ≤ 6 at 5 min, need for FiO_2_ ≥ 0.4 for resuscitation in the delivery room and pH ≤ 7.20 on umbilical vein samples. Moreover, AOPP cord blood levels higher than 90.70 μmol/L showed a significant association with IVH development in a small preterm cohort [88]. CSF concentration of AOPP in preterm infants has also been investigated; subsequent evidence of white matter injury on term MRI resulted associated with significantly higher AOPP levels compared to normal MRI [84]. Overall, these findings may suggest an increased vulnerability of the preterm population to protein oxidation.

### 3.3. Nucleic Acid Oxidation Markers

The oxidized DNA nucleoside 8-hydroxydeoxyguanosine (8-OHdG) results from DNA peroxidation, and its concentration on urine and serum samples has been proposed as a biomarker for oxidative changes to nucleic acids in the neonatal population [89,90,91]. Fukuda et al. examined urinary and CSF levels of 8-OHdG to estimate the relevance of oxidative stress in infants with different types of brain damage. Increased urinary 8-OHdG levels were observed in neonates with HIE and SNC infections compared to healthy controls; in a similar vein, 8-OHdG concentration in the CSF of the HIE and SNC infection groups was 2 and 3 times higher than controls, respectively, supporting the role of oxidative stress in the acute brain injury associated with these pathological conditions [92].

### 3.4. Antioxidant Enzymes

CAT, GP and SOD are the main antioxidant enzymes involved in the first defense from oxidative stress and, as such, have been investigated as possible biomarkers for oxidative brain injury in at-risk neonates.

Increased levels of these three enzymes on cord samples and on plasma specimens obtained during the first 24 h have been described in asphyxiated term neonates compared with healthy controls [68,71,72,83]. With regard to the association with HIE severity, day-1 concentration of plasmatic SOD was significantly higher in infant with moderate or severe HIE compared to mild or no HIE [71,83], but did not differ significantly between moderate and severe cases [83]. On the other hand, plasmatic CAT increased at increasing Sarnat stages, with significant differences within mild, moderate and severe HIE, and was significantly higher in infants who survived compared to those who did not [83]. These results, together with the evidence of a significant correlation between SOD levels and serum lipid peroxides [71], are consistent with the up-regulation of antioxidant capacities in response to the oxidative burst ensuing from the hypoxic-ischemic insult. In CSF specimens obtained from HIE infants during the first 72 h of life, GP and CAT activity were significantly increased only in those with severe HIE compared to mild and no HIE, while SOD activity was significantly higher in the whole HIE group compared to controls, but did not differ within different HIE stages [93].

Conversely, despite significantly increased superoxide levels, no difference in SOD activity was observed in the cord blood of a small cohort of preterm infants developing early PVL compared to those with no evidence of brain injury [94]. This is consistent with the developmental lag of SOD expression observed before near-term age, which may contribute to increase the risk for PVL in the preterm population [3]. These data suggest that, when evaluated in preterm infants, antioxidant enzymes may be unreliable to assess their redox status.

### 3.5. Non-Protein-Bound Iron (NPBI)

By supplying the availability of redox-cycling iron, the release of intracellular NPBI following hypoxic-ischemic or hemorrhagic insults enhances oxidative stress. Being rich in iron, which is required for their differentiation, pre-OL are a vulnerable target of NPBI-mediated damage [57]. Moreover, plasma NPBI may contribute to oxidative brain injury by leaking into the cerebral tissue through a damaged BBB. Elevated NPBI levels in a mixed preterm and term neonatal population has been shown to significantly correlate with isoprostanes [95] and PC [96], thus underscoring the noxious oxidative effects of this highly reactive compound on biological structures. After adjustment for GA, Apgar score, cord pH and base excess, a NPBI concentration > 15.2 mmol/l on cord blood was found to effectively predict poor neurodevelopment at 24 months in a large cohort of both term and preterm infants [97].

Plasma NPBI following perinatal asphyxia were first evaluated more than 20 years ago by Dorrepaal et al., who observed increasing percentages of detectable NPBI with increasing HIE severity in asphyxiated infants > 34 weeks’ gestation; in this study, NPBI concentration within the first 8 h had the most significant predictive value towards 12 month-neurodevelopment [98]. Similarly, later studies reported significantly increased plasma [69,74] and CSF levels [69] within the first 72 h of life in term neonates with moderate or severe HIE, with higher levels in those who developed long-term neurological sequelae.

With regard to prematurity-related complications, a significant association between cord blood levels of NPBI and the development of all grades of IVH was observed, and a cut-off value of 10.07 μmol/L has been proposed to identify infants at higher IVH risk [88]. Following IVH, CFS levels of NPBI in preterm infants with posthemorrhagic ventricular dilatation (PHVD) have also been evaluated [99]. NPBI was detected in 75% of PHVD infants, but in none of those in the control group; within the PHVD group, however, NPBI levels did not correlate with parenchymal brain lesions, need for shunt surgery and long-term disability [99].

### 3.6. Uric Acid

Using xanthine or hypoxanthine as reaction substrates and O_2_ as a cofactor, XO generates uric acid and O_2_^•−^, which further enhances free radical production [15]. Differently from the oxidative biomarkers previously described, uric acid concentration can be easily determined with long-stand, simple and largely available techniques (e.g., uricase-peroxidase method); hence, serving as a proxy for XO activity, serum and urinary levels of uric acid may represent a low-cost and easily accessible marker for oxidative stress in neonatal settings.

With regard to hypoxic-ischemic injury, current evidence is largely consistent in reporting an increased ratio between urinary uric acid and urinary creatinine (uUA/uCr) in both term and late preterm newborns with perinatal asphyxia compared to controls within the first 48–72 h of life [62,63,100,101,102,103]. This ratio showed a significant correlation with Sarnat staging [62,104], with increasing UA levels for increasing HIE severity. A cut-off level of 2.3 has been proposed to predict mortality in asphyxiated term infants [62,103].

On the other hand, the relationship between plasma UA and the development of severe IVH or PVL in preterm neonates is controversial. Pearlman et al. reported increased UA concentrations on the first postnatal day in ELBW infants developing severe IVH/PVL, even after adjustment for GA and other relevant clinical variables [105]; subsequent data from a larger sample of preterm neonates with similar brain injury, however, failed to confirm this association [106,107]. Finally, significantly higher UA levels were observed in the CSF of preterm neonates with IVH grade 2 to 4 [107].

### 3.7. Nitric Oxide

Due to the up-regulation of NOS expression that follows hypoxic-ischemic insults, the production of NO is enhanced and may trigger an overproduction of peroxynitrite and other RNS. Consistently, an increased concentration of NO and a higher nitrate/nitrite ratio have been detected in blood samples obtained within the first 24 h in neonates with HIE compared to controls [70,71,108,109], with remarkably higher levels in those with neuroradiological evidence of brain damage [108] and severe neurological compromise [71,108,109]. NO concentration in the CSF of asphyxiated babies was also investigated, showing significant increases in the most severe cases within the first 24 h after the hypoxic-ischemic hit [109].

### 3.8. Other Free Radical Biomarkers

Xanthine and hypoxanthine are the substrate for the oxidative reaction that leads to UA and O_2_^−^ formation. The concentration of these molecules in the cord blood of hypoxic and normoxic preterm infants has been investigated by Buonocore et al., who observed higher levels in the hypoxic subgroup [86]. In their study, xanthine and hypoxanthine concentration increased in a linear fashion with the severity of hypoxia and significantly correlated with the levels of total hydroperoxide, lipid and protein oxidation products.

Melatonin acts as a free radical scavenger and enhances antioxidant defenses; for this reason, serum melatonin has been evaluated as a possible oxidative biomarker in relation to brain injury. Evidence from the neonatal population is limited to Yan & Zhang [110], who reported a significant correlation between serum melatonin concentration and the severity of such brain lesions as IVH, PVL or cerebral infarction among preterm neonates. This finding may reflect an up-regulation of endogenous melatonin production in response to the noxious mechanisms underlying brain injury development.

## 4. Antioxidant Neuroprotective Treatments

Following the growing amount of evidence on the role of free radicals in the development of neonatal brain injury, new treatment perspectives aimed at counteracting the oxidative burden have emerged for term and preterm neonates at high neurological risk. While the neuroprotective effects of several therapeutic approaches have already been investigated in clinical settings, other molecules (e.g., edaravone, resveratrol, deferoxamine, NOS inhibitors) have been investigated only in preclinical studies, yielding promising preliminary results [111].

In this review, we will selectively focus on the neuroprotective strategies based on antioxidant mechanisms of action for which clinical evidence is currently available. These strategies contribute to reduce the oxidative burden through different mechanisms of action; hence, as illustrated in Figure 1, they may have disease-specific indications, depending on the pathophysiology of brain injury. The applicability of these approaches is further defined by their characteristics, such as the safety profile or the ability to cross the placental interface, which opens the way to antenatal treatment perspectives.

Among the included strategies, only two have become part of the standard care to prevent neonatal brain injury (i.e., TH for HIE and maternal magnesium sulfate for prematurity-related brain damage), while other pharmacological treatments, such as erythropoietin, have been approved for other indications in the neonatal population, and phase III trials evaluating their neuroprotective effects are currently ongoing. Further clinical evidence is required to support the potential benefits against the development of neonatal brain injury of less extensively investigated molecules, such as melatonin and N-acetylcysteine.

### 4.1. Therapeutic Hypothermia

TH, started within the first six hours after delivery and maintained at 33.5 °C for 72 h, is the treatment of choice for HIE in term and late-preterm infants [112]. TH down-regulates cerebral energy metabolism, thus subsequently decreasing the secondary energy failure phase that follows the acute hypoxic-ischemic insult and the ensuing apoptotic burden [113,114]. The slight reduction in tissue O_2_ supply that has been reported during mild hypothermia, as a possible result of the increased hemoglobin O_2_ affinity at lower temperatures, may contribute to limit the oxidative burst triggered by the increased O_2_ availability that follows reperfusion [115]. In addition, by increasing ATP stores and slowing the activity of ion channels, TH contributes to maintain the integrity of neuronal membranes and to subsequently decrease glutamate excitotoxicity [116,117]. A diminished iNOS transcription has been observed during TH, as a possible consequence of the inhibition of the NFκB pathway [118], thus decreasing the NO-mediated production of cyclic GMP in the cortex and in the nucleus striatum [119,120]. Evidence from an animal HIE model has also shown protective effects of mild TH on pre-OL maturation and differentiation [121]. Consistently with these mechanisms of action, a decreased severity of basal ganglia or thalamic lesions and of white matter injury were observed in HIE neonates undergoing moderate TH [122,123].

The safety and efficacy of TH in reducing the risk of death and major neurodevelopmental impairment in neonates with moderate to severe encephalopathy has been widely established in multiple clinical trials and confirmed by several meta-analyses [124,125,126,127]; as such, TH has widely become the standard of care for term and near-term neonates following perinatal asphyxia [17]. To date, however, there is insufficient data to extend the use of TH in asphyxiated preterm infants below <35 weeks’ gestation, despite the highest neurological risk of this population. The most relevant matter of concern is safety, since current literature has reported significantly increased rates of mortality and adverse events (e.g., acidosis, hypotension, leukopenia, coagulopathy, hyper- or hypoglycemia) when TH was applied at lower GA ranges [128,129]. Hence, while awaiting further data on TH, it is important to validate alternative neuroprotective strategies to limit the burden of brain injury in the preterm population.

Moreover, in low-income countries, TH is not ubiquitously available, or may be associated with increased adverse effects compared to supportive care, with a paradoxical increase of mortality rates and lack of short-term neurological benefits [130]. Hence, the development of neuroprotective strategies easily available at low cost, with a strong safety profile or endowed with neuroregenerative properties may aid to reduce the burden of HIE-related brain injury in these settings.

### 4.2. Erythropoietin

Erythropoietin (Epo) is an erythropoiesis-stimulating hormone which is also endowed of cytoprotective effects in non-hematopoietic tissues, including the brain. By binding to its receptors, which are largely expressed in the CNS, Epo exerts its neuroprotective properties by activating antioxidant and anti-inflammatory signaling pathways, which stimulate neurogenesis and lead to a downstream reduction of neuronal apoptosis [131]. In vitro studies investigating Epo-related antioxidant effects on the brain tissue have shown a suppressed ROS production in microglial cells [132], restoration of GP activity in substantia nigra and astroglial cells [133], reduced brain levels of lipid peroxidation products [134] and of NO [135] following Epo administration.

Due to its relatively large size, however, only a minor percentage can cross the BBB; hence, compared to erythropoietic indications, higher doses are required to increase Epo concentrations in the CNS. Recombinant human erythropoietin (rh-Epo) has been approved for anemia treatment even in the neonatal population.

Hypoxia induces a significant up-regulation of both Epo secretion and Epo-R expression; while the latter increases promptly, the former rises with some latency [136]. The time interval during which the enhancement of endogenous Epo secretion is not fully achieved represents a useful therapeutic window for the potential Epo benefits in neonatal HIE [136]. According to currently available literature, HIE neonates undergoing Epo treatment, either in monotherapy [137,138,139,140] or combined to therapeutic hypothermia [141,142,143], have less abnormalities at neuroimaging [138,144], fewer seizures [138,141], a lower incidence of cerebral palsy and of moderate to severe disability [137,138,143] and better psychomotor outcomes up to 24 months of age [137,138,139,142,143]. However, since many of the available studies are either based on underpowered cohorts, or did not entail a randomized intervention or a control group, further data from ongoing randomized double-blind control trials (NCT02811263; NCT03079167) are expected.

The neuroprotective effects of rh-Epo on prematurity-related neurological complications have been investigated in a recent metanalysis, which showed significantly lower incidence of severe IVH (i.e., grade III-IV) and of PVL in treated preterm neonates [145]. Moreover, in two randomized trials where preterm infants received high-dose rh-Epo versus placebo, significantly decreased white matter abnormalities, periventricular white matter loss and improved white matter development at term MRI were observed in the treated group [146,147]. In very preterm infants with IVH, rh-Epo administration following IVH diagnosis was associated with significantly lower rates of death, neurological disability and severe neurodevelopmental impairment at 18 months compared to placebo [148]; the results of a large double-blind, placebo-controlled trials, however, are expected to further confirm this data [149].

Despite the encouraging evidence discussed above, attention should be paid to the possible adverse effects associated with Epo treatment. While no difference in adverse outcomes or Epo-related complications, including hematological effects, have been reported in treated HIE neonates compared to untreated ones [150], the higher Epo doses required to cross the BBB may increase the risk of adverse effects in the preterm population, where significantly higher hematocrit, reticulocyte, and white blood cell counts and lower platelets following Epo treatment have been reported [151]. An additional matter of concern related to Epo administration in the preterm population was the possible development of retinopathy of prematurity; according to current literature, however, Epo treatment does not increase the incidence of this condition in preterm infants [145]. Alternative Epo analogs that may enhance Epo transport through the BBB or prolong its half-life, allowing to use lower doses, have been developed. Darbepoetin is a modified long-acting Epo analog that has been investigated in neonatal clinical settings [152,153,154]; current data, however, do not allow to draw conclusions on darbepoetin use for neonatal neuroprotection [145], and a phase II trial on mild HIE infants is currently recruiting (NCT03071861).

### 4.3. Melatonin

Melatonin is an indoleamine hormone produced by pineal gland, primarily involved in sleep-wake cycle regulation [155], but also endowed with antioxidant, anti-inflammatory and anti-apoptotic properties. Among its antioxidant effects, melatonin acts as a direct ROS scavenger, enhances ETC efficiency and the enzymatic activity of SOD, GP and glutathione reductase [156] and, by dampening iNOS expression, reduces the subsequent RNS formation [157]. Due to its lipophilic features, melatonin can easily cross the BBB, and has thus been proposed as a possible neuroprotective agent.

The antioxidant benefits of melatonin as well as its safety in the context of neonatal HIE are supported by robust preclinical evidence; however, currently available data from human trials are limited [158,159]. Fulia et al. reported significantly lower levels of serum MDA and nitrite/nitrate ratio, and reduced mortality rates in a small cohort of asphyxiated neonates receiving melatonin treatment [158]. More recently, melatonin administration in asphyxiated infants during hypothermia resulted in significantly enhanced SOD activity, diminished serum NO levels, fewer seizures, reduced evidence of white matter injury on MRI and improved survival without neurological or developmental impairment at 6 months [159]. Preliminary results of an ongoing clinical trial (NCT02621944) suggest the preferential use of the intravenous route for melatonin administration to achieve 100% bioavailability, and have shown that melatonin pharmacokinetics are not altered by therapeutic hypothermia [160]. Larger randomized controlled trials are required to support this long-awaited treatment to reduce brain injury in following neonatal HIE.

Despite the availability of promising preclinical findings, the neuroprotective effects of melatonin on white matter injury and IVH have not yet been established in clinical settings.

### 4.4. Allopurinol

Acting as a XO activity inhibitor and scavenging hydroxyl free radicals and NBPI, allopurinol represents a possible therapeutic strategy against oxidative tissue damage. Given the ability of allopurinol to cross the BBB, several clinical studies have currently investigated the neuroprotective potential of allopurinol in relation to hypoxic-ischemic neonatal brain injury. Van Bel et al. [161] reported no increase in serum MDA concentration, significantly lower serum levels of NPBI and uric acid and a more stable electrical brain activity in HIE neonates treated with high-dose (40 mg/kg) allopurinol within the first 4 h from resuscitation, compared to untreated ones. Similar allopurinol doses significantly decreased NO levels in serum, but not CSF samples, in treated asphyxiated infants at 72–96 h of life [109]. In cases of severe asphyxia, however, allopurinol failed to reduce HIE-related mortality and morbidity [162]; in addition to the disease severity, the prolonged time interval (up to 4 h) elapsed between ischemia-reperfusion and treatment initiation was brought up by the authors as a possible cause for the observed findings. Follow-up data on long-term neurodevelopmental outcomes following allopurinol treatment are controversial: while Gunes et al. [109] reported better outcomes at ≥12 months of age in treated infants compared to untreated controls, at 5-year follow-up a less severe impairment was observed only in the moderate HIE subgroup, but not in the whole HIE cohort [163].

The ability to cross also the placental interface has led to investigate the neuroprotective potential of peripartum allopurinol administration in the presence of fetal hypoxia (e.g., abnormal/non-reassuring fetal heart rate tracing, evidence of acidosis at scalp pH). Following the first clinical evidence of significantly reduced NPBI and brain injury markers in neonates born from treated mothers [164], a randomized, double-blind, placebo-controlled multicenter trial (ALLO-trial) was designed to investigate the efficacy of maternal allopurinol administration against HIE development [165]. According to the ALLO-trial results, intravenous administration of 500 mg of allopurinol in laboring women had an adequate safety profile in mothers and neonates and allowed the achievement of target fetal concentrations within 5 min from the end of the infusion [166]. However, the evaluation of brain damage markers showed no significant difference between treated and untreated newborns, although a potential gender-related effect was hypothesized by the evidence at the post-hoc analysis of lower S100ß and neuroketal cord levels in female neonates born from treated mothers [167].

In sum, available clinical evidence on the neuroprotective properties of antenatal or postnatal allopurinol in neonatal HIE is still inconclusive [168]; results from ongoing randomized controlled clinical trials (NCT03162653; http://www.albino-study.eu, accessed on 16 December 2021) might help to clarify the usefulness of this treatment against hypoxic-ischemic neonatal brain damage.

Evidence on allopurinol-related neuroprotection towards prematurity-related brain injury is very limited [169]. Despite PVL development was preceded by significantly higher levels of oxidative biomarkers in cord blood, prophylactic allopurinol administration in preterm infants between 24 and 32 weeks’ gestation failed to significantly reduce the incidence of PVL compared to placebo [169]. After this study, however, allopurinol treatment as a neuroprotective strategy in the preterm population has not been further investigated.

### 4.5. N-Acetylcysteine

N-acetylcysteine (NAC) is a membrane-permeable cysteine precursor that acts as a ROS scavenger, replenishes cellular GSH [170] and down-regulates iNOS expression [171]. Due to these antioxidant characteristics, together with its ability to cross the BBB and its low toxicity, NAC is a high-potential candidate for neonatal neuroprotection.

Current clinical evidence has demonstrated that NAC infusion rapidly and significantly increases GSH levels in basal ganglia, evaluated by magnetic resonance spectroscopy at 5 days of life in a small cohort of HIE neonates who had previously undergone TH [172]. Given the feasibility of NAC placental transfer, the neuroprotective effects of antenatal and postnatal NAC treatment in newborns exposed to chorioamnionitis were investigated [173]. Compared to placebo, treated infants showed an improved cerebrovascular regulation; additional results on clinical and neuroradiological outcomes, however, have not been published yet [173].

With regard to white matter injury or IVH, current evidence has failed to demonstrate any beneficial effect of NAC in reducing the incidence of these conditions among treated preterm neonates, primarily due to underpowered sample sizes [82,174]; hence, larger targeted studies are warranted to ascertain the possible NAC effectiveness in reducing prematurity-related brain injury.

### 4.6. Magnesium Sulfate

Magnesium ion is an NMDA receptor blocker that, among its biological effects, prevents glutamate excitotoxicity, inhibits the activation of inflammatory pathways and has also been associated with reduced oxidative damage [175,176,177], with relevant implications in relation to brain injury development.

Thanks to its low cost and wide availability, the neuroprotective potential of magnesium sulfate has been largely investigated in clinical trials on pregnant women at risk for preterm birth. As previously established in 2009 [178,179] and further confirmed by more recent metanalyses inclusive of the latest trials [180,181], magnesium infusion prior to preterm delivery effectively decreased the postnatal incidence of cerebral palsy, which typically develops following white matter injury, regardless of the reason for preterm birth, with similar effects across a range of preterm gestational ages and different treatment regimens. This extensive clinical evidence supporting the beneficial effects of maternal magnesium administration on neonatal motor outcomes at 2 years, the low-toxicity profiles of magnesium sulfate and its highly favorable cost-effectiveness ratio has led, over the past years, to include this treatment as a standard of care in women at risk for preterm delivery. Neurodevelopmental follow-up data at school-age, however, failed to demonstrate significant differences in motor, cognitive and behavioral outcomes between previously treated vs. untreated children [182,183].

In term infants, the neuroprotective effects of antenatal magnesium administration have been investigated in the context of mild pre-eclampsia with evidence of fetal distress, failing to demonstrate specific neurological benefits associated with antenatal magnesium administration [184]. However, the relatively small cohort, as well as the increased resistance of term infants to oxidative stress and the relatively mild oxidative effects associated with mild pre-eclampsia may have contributed to these non-significant results.

With regard to hypoxic-ischemic brain injury, clinical evidence is inconsistent. To date, several randomized placebo-controlled trials have investigated the neuroprotective potential of magnesium sulfate administration in asphyxiated term neonates. In 2013, a metanalysis of the available literature showed a significant reduction of abnormalities at neurological examination or neuroimaging, but failed to prove magnesium efficacy in preventing death, seizure development and moderate-to-severe neurodevelopmental disability at age 18 months [185]. Later evidence from low-income settings, where magnesium sulfate would represent a potentially viable therapy due to its low cost and ease of delivery, failed to prove substantial developmental and neuroradiological benefits following magnesium sulfate administration in neonatal HIE [186,187], despite reported improvements in seizure burden and neurological examination at discharge [188]. The lack of substantial effectiveness of magnesium sulfate in relation to neonatal HIE brings the following considerations. First, predicting HIE development prior to birth is a challenge, therefore in HIE studies this treatment was commenced within 6 h after the noxious insult, while magnesium sulfate for preterm infants’ neuroprotection is administered antenatally. Moreover, postnatal treatment was associated with TH only in a small number of studies, potentially contributing to the heterogeneity of the observed results. Useful information may be obtained from translational preclinical models of perinatal encephalopathy, before large randomized clinical trials on magnesium sulfate for HIE are undertaken [189].

## 5. Conclusions

The growing amount of evidence on the crucial role of oxidative stress in the development of neonatal brain injury has provided a deeper understanding of the underlying pathophysiological mechanisms that lead to the noxious free radical overproduction. Depending on these different mechanisms of damage and on specific neonatal characteristics, such as GA and its maturational effects on antioxidant defenses and cerebrovascular autoregulation reflexes, different profiles of brain injury can be identified. Based on this evidence, multiple biomarkers aimed at estimating the burden of the ongoing oxidative processes have been explored over the past decades, yielding promising results and showing a good correlation with the clinical status. However, despite their high potential, these biomarkers require a more extensive validation on larger trials before their use in routine neonatal care can be considered. At the same time, several treatments aimed at reducing oxidative stress have been assessed as possible neuroprotective strategies. Two important treatments endowed with antioxidant properties, such as therapeutic hypothermia and magnesium sulfate, are now part of the standard of care to reduce the risk of neonatal brain injury, while other promising therapeutic strategies are currently being examined in phase 1 to 3 clinical trials. The implementation of this currently available evidence is of paramount importance to develop tailored diagnostic and therapeutic approaches in order to limit the oxidative burden on the developing brain and to further improve neonatal neurological outcomes.

## Figures and Tables

**Table 1:** Clinical evidence on free radical biomarkers in relation to hypoxic-ischemic encephalopathy (HIE) development and subsequent neurological and developmental sequelae.

Biomarkers		Plasma/Serum	Urine	Cerebro-Spinal Fluid
**Lipid** **peroxidation**	*Malondialdehyde*	Increased cord blood levels in asphyxiated infants [63,67] Increased levels in HIE infants at 0–72 h [67,68,69,70,71,72]; significant correlation with HIE severity [63,68,71] Highest levels in non-survivors [67,69], in the presence of brain lesions at neuroimaging [73] or in case of persistent neurological impairment [69,74]	Increased levels in asphyxiated neonates [62,63,64] Significant correlation with HIE severity and mortality [62,75]	Increased levels in asphyxiated infants developing HIE [69,83] Higher levels in infants with persistent neurological impairment or non-survivors [69]
	*Isoprostanes*	Increased cord blood levels in asphyxiated infants [79] *8-isoprostane*: no significant correlation with HIE severity or evidence of brain lesions at MRI [80]	Not available	Not available
**Protein** **oxidation**	*Protein carbonyls* (*PC*) *Advanced oxidation protein products* (*AOPP*)	Increased PC levels in HIE infants; higher levels if seizures development [67] Trend towards increased AOPP levels (not significant) in term HIE infants [68]	Not available	Not available
**Nucleic acid oxidation**	*8-hydroxy-2-deoxyguanosine*	Not available	Increased levels in HIE infants [92]	Increased levels in HIE infants (2-fold) [92]
**Antioxidant** **enzymes**	*Superoxide dismutase* (*SOD*) *Glutathione peroxidase* (*GP*) *Catalase* (*CAT*)	Increased SOD, GPT, CAT levels at 0–24 h in asphyxiated term neonates [68,71,72,83] SOD and CAT (0–24 h): correlation with HIE severity [71,83]	Not available	Increased SOD activity in HIE neonates [93] Increased GP and CAT activity in severe vs. mild/no HIE [93]
**Non-protein-bound iron (NPBI)**		Increased levels in HIE infants [69,98]; correlation with HIE severity [98] 0–8h levels may predict 12-month neurodevelopment [98] Trend towards increased levels (not significant) in asphyxiated neonates with persistent neurological impairment [74]	Not available	Increased levels in HIE infants [69]
**Uric acid (UA)**		Not available	Increased UA levels in term and preterm asphyxiated neonates [62,63,100,101,102,103] Correlation with HIE severity [62,104] UA/urine creatinine ratio > 2.3 may predict mortality in term HIE infants [62,103]	
**Nitrosative** **biomarkers**	*Nitric oxide* (*NO*) *Nitrate/nitrite ratio*	Increased NO and nitrate/nitrite ratio in HIE neonates [70,71,108,109] Higher levels associated with evidence of brain lesions [108] and severe neurological impairment [71,108,109].	Not available	Increased NO at 0–24 h in severe HIE [109]

**Table 2:** Clinical evidence on free radical biomarkers in relation to the development of preterm infants’ brain injury and subsequent neurological and neurodevelopmental sequelae. Abbreviations: IVH, intraventricular hemorrhage; PHVD, post-hemorrhagic ventricular dilatation; PVL, periventricular leukomalacia.

Biomarkers		Plasma/Serum	Urine	Cerebro-Spinal Fluid
**Lipid** **peroxidation**	*Malondialdehyde* (*MDA*)	Increased in preterm infants who developed IVH [76]	Conflicting results in relation to IVH/PVL development [77,78]	Not available
	*Isoprostanes*	Correlation between 24–48 h levels and white matter injury severity at term MRI [81]; Increased 8-isoprostane levels on day 3 and 7 in infants with severe IVH [82] Increased 8-isoprostane levels on day 7 in infants who developed PVL [82]	Not available	Trend toward higher 8-isoprostane levels (not significant) in infants developing white matter injury [84]
**Protein** **oxidation**	*Advanced oxidation protein products* (*AOPP*)	Increased IVH risk for cord blood AOPP > 90.70 μmol/L [88]	Not available	Positive correlation between AOPP levels and white matter injury severity at term MRI [84]
**Nucleic acid oxidation**	*8-hydroxy-2-deoxyguanosine*	Not available	Not available	Not available
**Antioxidant** **enzymes**	*Superoxide dismutase* (*SOD*) *Glutathione peroxidase* (*GP*) *Catalase* (*CAT*)	No difference in cord blood SOD in preterm infants developing early PVL vs. controls [94]	Not available	Not available
**Non-protein-bound iron (NPBI)**		Increased cord blood levels in infants who developed IVH (all grades) [88] Increased IVH risk for cord blood NPBI > 10.07 μmol/L [88]	Not available	Detectable levels in 75% of PHVD infants (vs. 0% in the control group) [99] No correlation with parenchymal brain lesions, need for shunt surgery, long-term disability [99]
**Uric acid (UA)**		Conflicting data on the association with severe IVH/PVL [105,106,107]	Not available	Increased levels in preterm infants with IVH grade 2–4 (mean age: 8 days) [107]
**Melatonin**		Increased melatonin levels in preterm infants with IVH, PVL or cerebral infarction (cut-off value: 69.5 pg/mL) [110] Correlation with brain injury severity [110]	Not available	Not available
